# Ethylene oxide exposure increases impaired glucose metabolism in the US general population: a national cross-sectional study

**DOI:** 10.1265/ehpm.24-00199

**Published:** 2024-12-07

**Authors:** Yuqi Zhao, Deliang Liu, Xiaogao Pan, Yuyong Tan

**Affiliations:** 1 Department of Gastroenterology, Second Xiangya Hospital, Central South University, Changsha 410011, China; 2 Research Center of Digestive Disease, Central South University, Changsha 410011, China; 3 Clinical Research Center for Digestive Disease in Hunan Province, Changsha 410011, China; 4 Department of Emergency Medicine, Second Xiangya Hospital, Central South University, Changsha 410011, China; 5 Emergency Medicine and Difficult Diseases Institute, Central South University, Changsha 410011, China

**Keywords:** Glucose metabolism, Diabetes mellitus, Prediabetes, Ethylene oxide

## Abstract

**Background:**

Current experimental evidence supports that ethylene oxide (EO) exposure-related pathophysiologies may affect glucose metabolism, but few population-based studies have explored the potential links.

**Methods:**

This study used cross-sectional data from 15560 participants in the National Health and Nutrition Examination Survey (NHANES) from 2017 to 2020. EO exposure levels were calculated by testing hemoglobin adducts of EO (HbEO) via a modified Edman reaction. We focused on the association of EO exposure with prediabetes and diabetes as well as indicators of impaired glucose metabolism and further analyzed the potential pathogenic mechanisms. Statistics included logistic regression, generalized additive model fitting, penalized spline method, two-piecewise linear regression, recursive algorithm, mediation analysis, and Pearson’s analysis.

**Results:**

EO exposure was associated with changes in glucose metabolic indicators and increased prevalence of prediabetes and diabetes, showing age-consistency and being more pronounced in obese and non-smoking populations. For each one pmol/g Hb, one SD, or two-fold SD increase in log2-HbEO, the risk of prediabetes increased by 12%, 16%, and 33%, with an increased risk of diabetes by 18%, 26%, and 61%, respectively. Dose-response curves revealed that this positive correlation was approximately linear with prediabetes and “J” shaped with diabetes. When log2-HbEO > 8.03 pmol/g Hb, the risk of diabetes would be further increased. Pearson’s correlation revealed that EO exposure was associated with reduced fasting insulin and elevated HbA1c in the prediabetic stage. While in the diabetes stage, EO exposure was correlated with elevated fasting glucose, HbA1c, and HOMA-IR, suggesting an exacerbation of diabetes progression by EO exposure. A potential mechanism that the early stages of impaired glucose metabolism may be initiated by EO-related inflammation and oxidative stress damaging pancreatic β-cells, resulting in decreased insulin secretion. These speculations were partially supported by mediation analysis and mediators’ Pearson analysis.

**Conclusion:**

Elevated ethylene oxide exposure increases the incidence of impaired glucose metabolism in the general U.S. population and a potential intervention may be to effectively suppress inflammation and oxidative stress imbalances.

**Supplementary information:**

The online version contains supplementary material available at https://doi.org/10.1265/ehpm.24-00199.

## Graphical Abstract

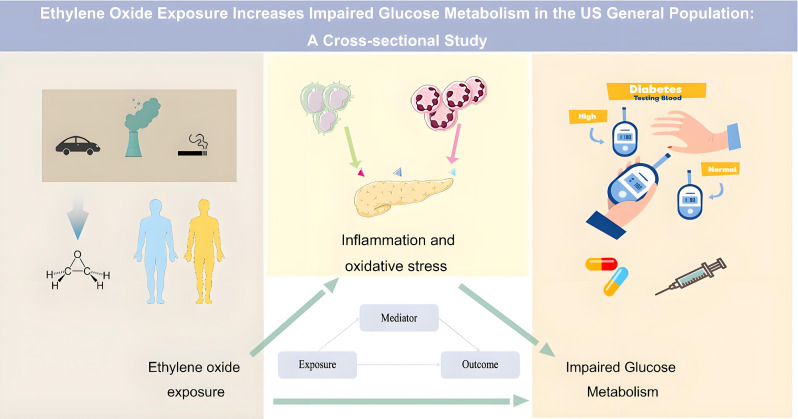

## Introduction

Ethylene oxide (EO) (chemical formula C2H4O) is a highly reactive alkylating gas that is widely used in the sterilization of food and medical devices and the manufacture of household products [1]. With an annual global production of nearly 35 million tons, it is one of the most produced organic chemicals [2]. Pharmacokinetic data reveal that EOs are readily absorbed into the respiratory tract of humans and other animals through sterilized medical devices, fumigated food and spices, inhalation of contaminated air, tobacco smoke, or vehicle exhaust [1, 3]. After entering the body, EO alkylates proteins and DNA and is metabolized primarily by non-enzymatic hydrolysis, enzymatic hydrolysis, and glutathione conjugation [1]. Multiple experiments in vitro and vivo have established a link between EO exposure and a range of harmful health effects, such as cancer [4, 5], genotoxicity [6], neurological damage [7, 8], and systemic inflammation [9, 10]. As a result, EO has been classified as carcinogenic to humans (Group 1) by the International Agency for Research on Cancer and the U.S. Environmental Protection Agency [11]). This has led to a high public concern about the potential adverse health effects of EO exposure. The evaluation regarding EO exposure levels and potential disease risks becomes important for health management decisions.

Diabetes mellitus is a chronic disease that poses a serious threat to human health and is the leading cause of various cardiovascular events and neurological dysfunction. According to the International Diabetes Federation, the population of adults with diabetes has reached 537 million worldwide, accounting for about one-tenth of the global adult population, and it has become a major global health burden. By 2050, the world’s population with diabetes is projected to increase to 1.3 billion [12]. There will be a significant increase in mortality and the socio-economic burden of diabetes. Prediabetes, the intermediate stage between normal blood glucose regulation and diabetes, is associated with a high risk of diabetes and its complications, with approximately 10% of people with prediabetes developing diabetes each year [12]. Currently, 720 million adults worldwide are affected by prediabetes, which is projected to reach more than one billion by 2045 [12]. The growing prevalence presents a significant societal challenge to the health of all races and age groups.

Multiple mechanisms of pancreatic β-cell dysfunction and insulin resistance, such as genetic susceptibility, chronic inflammation, immune dysregulation, and pancreatic amylin deposition, are important contributors to impaired glucose metabolism with subsequent diabetes progression [13]. Based on current evidence that excessive EO exposure could increase genotoxicity [6], decrease levels of the antioxidant glutathione [14], induce inflammation, and develop immune dysregulation [9]. These EO exposure-related pathophysiologies are potentially linked to the development of impaired glucose metabolism [15, 16]. In addition, this link has been indirectly tested in population-based studies, such as Xie et al., who found that EO exposure was closely related to the “ Life’s essential 8” score, including blood glucose [17]. Other reports also found similar interactions with diabetes, such as cardiovascular disease, metabolic syndrome, and hypertension [18–20]. Such a link provides the possibility that there is an association between EO exposure levels and prediabetes (an early stage of diabetes) and diabetes. Previously Guo et al. reported an association between EO exposure and the prevalence of diabetes [21]. However, they focused on trends in the effects of EO exposure from tobacco smoke on diabetes and lacked analysis about prediabetes as well as more detailed information. Prediabetes and diabetes are different stages of glucose metabolism dysfunction, where intervention in prediabetes can more effectively minimize the prevalence and onset age of diabetes. Consequently, we focused on the relationship between EO exposure and these two diseases as well as indicators of impaired glucose metabolism, and further analyzed the potential pathogenic mechanisms. It is hoped that these findings could provide a timely warning to sensitive individuals about the risk of such health impairments, which would contribute to the improvement and management of glucose metabolism globally.

## Methods

### 1. Study population

The National Health and Nutrition Examination Survey (NHANES) is a national survey that monitors the health and nutritional status of adults and children across the United States. The current study is a cross-sectional analysis of data from NHANES 2017–March 2020 pre-pandemic (https://www.cdc.gov/nchs/nhanes/index.htm). During this period, 15,560 participants’ information was successfully collected, but only 3,637 participants had complete and qualified test data for EO exposure. After excluding participants with missing diabetes questionnaires, fasting glucose, and hemoglobin A1c (HbA1c), as well as those under 18 years old, data from the final remaining 3200 eligible participants were used for the analysis (shown in Supplementary Material Figure S1).

### 2. Assessment of HbEO

The level of EO exposure by all routes was calculated by testing hemoglobin adducts of EO (HbEO) in human whole blood or erythrocytes by modified Edman reaction. Specifically, the reaction products with the N-[2-hydroxyethyl]valine (HEV) of the hemoglobin protein chains are measured [22]. Then analyze Edman products by high-performance liquid chromatography coupled with tandem mass spectrometry (HPLC-MS/MS) and process results. The levels of HbEO were quantified and expressed in picomoles per gram of hemoglobin (pmol/g Hb). Detailed specimen collection and processing instructions are discussed in the 2017–2018 and 2019–2020 NHANES Laboratory Procedures Manuals (LPMs). For analytes with analytic results below the lower limit of detection (12.9 pmol/g Hb), the value of the lower limit of detection divided by the square root of 2 was placed in the analyte results field (9 pmol/g Hb). Based on available evidence, the half-life of HbEO is related to the life span of human erythrocytes (about 120 days), and its level reflects cumulative exposure to EO that has occurred in recent months [23, 24].

### 3. Ascertainment of prediabetes and diabetes

According to the criteria of the American Diabetes Association, diabetes mellitus was defined by self-reported diagnosis, use of insulin or oral hypoglycemic medication, fasting glucose ≥7.0 mmol/L, or HbA1c level ≥6.5%. Participants who met each one or more of the conditions listed above were recognized as diabetes mellitus. Prediabetes is identified by having fasting glucose between 5.6 mmol/L and 6.9 mmol/L, or HbA1c between 5.7% and 6.4% [25].

### 4. Covariates and mediators

We incorporated clinically meaningful covariates in this survey, including age, gender, body mass index (BMI), poverty income ratio (PIR), race and ethnicity, education status, smoking status, alcohol consumption, complete blood count, fasting glucose, fasting insulin, hemoglobin A1c (HbA1c), homeostatic model assessment of insulin resistance (HOMA-IR), alkaline phosphatase (ALP), γ-glutamyl transferase (GGT), hypertension, hemoglobin, triglycerides, and total cholesterol/high-density lipoprotein cholesterol (TC/HDL-C). All these variables are described on the NHANES website.

BMI was calculated as weight in kilograms (kg) divided by height in meters squared (m^2^). Race and ethnicity were classified as Mexican American, non-Hispanic white, non-Hispanic black, and others. Education status was classified as less than high school, high school graduate or equivalent, and some college or above. According to the smoking-cigarette use questionnaire, smoking status was classified as never smoking, ever smoking, and current smoker. According to the alcohol use questionnaire and the National Institute on Alcohol Abuse and Alcoholism criteria of heavy drink, which is >14 drinks (196 g) per week for males or >7 drinks (98 g) per week for females [26]. HOMA-IR was calculated by the formula: fasting insulin (µU/mL) × fasting glucose (mmol/L)/22.5. According to previous studies [9, 27], we selected ALP and GGT as markers of oxidative stress and white blood cells, neutrophils, lymphocytes, monocytes, and C-responsive proteins as markers to assess chronic inflammation, which has been widely utilized to measure oxidative stress and inflammation in NHANES studies. Hypertension was defined when the participants self-reported hypertension or used medication for hypertension. For those who did not have self-reported, according to the American Heart Association/American College of Cardiology (AHA/ACC) 2017 guideline for monitoring and diagnosis of hypertension. Participants with systolic blood pressure ≥130 mmHg or diastolic blood pressure ≥80 mmHg were also considered hypertension [28].

### 5. Statistical analysis

Continuous variables were expressed as mean ± standard deviation (SD) or median (IQR). Categorical variables were shown as percentages. Kruskal-Wallis test (skewed distribution), ANOVA (one way), chi-squared test (categorical variables), or t-test were used to analyze baseline features. In the case of count variables with theoretical values less than 10, Fisher’s exact probability test was employed. Blood HbEO levels were normalized by log2 conversion and divided into quartiles. When exploring the risk of prediabetes and diabetes, both are expressed relative to the euglycemia population, i.e., the dependent variable in the logistic regression is euglycemia vs. prediabetes or diabetes. To further explore whether ethylene oxide exposure correlated with impaired glucose metabolism in certain members, statistical analyses were done in three key steps.

First, logistic regression analysis was used to explore the single effect of EO exposure on the prevalence of prediabetes and diabetes, and three different covariate-adjusted models were constructed to show the associated risks. We designated the first quartile (Q1) as the control group and performed a linear trend analysis based on the log2-HbEO quartiles. Additionally, we stratified groups by age, sex, BMI, ethnicity, smoking status, and alcohol consumption to demonstrate sensitivities and interactions within different subgroups.

Second, generalized additive model fitting and penalized spline method (smooth curve) were used to explain the dose-effect response between EO exposure level and the prevalence of prediabetes and diabetes. In case any nonlinearity was observed, the inflection points were calculated utilizing a recursive algorithm. Next, a linear two-piecewise regression was constructed on both sides of the inflection point, and the best-fit model was determined based on the p-value of the likelihood log-ratio test. An adjustment was made for all covariates in this process to demonstrate the independent effect of EO exposure. Meanwhile, Pearson correlation analysis was employed to explore the relations between EO exposure and glucose metabolism indicators, including fasting glucose, fasting insulin, HbA1c, and HOMA-IR.

Third, based on the positive correlation established between ethylene oxide exposure and the prevalence of prediabetes and diabetes, we performed multiple linear regression analyses to explore the relationship between inflammation and oxidative stress with EO exposure and the prevalence of prediabetes and diabetes, respectively. To further examine potential pathogenic mechanisms as well as direct and indirect relationships, we performed a mediation analysis using nonparametric bootstrapping (n = 1000) to demonstrate the extent of the mediating effect after adjusting for all covariates [29]. Finally, we analyzed tendencies of inflammatory and oxidative stress biomarkers (white blood cells, neutrophils, lymphocytes, monocytes, ALP, GGT) with increasing EO exposure in different states of impaired glucose metabolism using Pearson’s correlation.

The statistical analyses for this study were performed via R, version 4.3.2 (R Foundation) and EmpowerStats (https://www.empowerstats.com, X&Y Solutions, Inc., Boston, MA). P ≤ 0.05 was considered statistically significant.

## Results

### 1. Population characteristics

The baseline characteristics of the log2-transformed HbEO (log2-HbEO) quartile subgroups are shown in Table 1, and those for impaired glucose metabolism are presented in Supplementary Table S1. Overall, participants had an average age of 45.36 ± 20.81 years, with a prevalence of 36.22% (1159 cases) for prediabetes and 15.19% (486 cases) for diabetes. Overexposure to EO was more common in males and more prevalent in non-Hispanic blacks, those with college or higher education, and smokers. Among the indicators of impaired glucose metabolism, impaired fasting glucose was not significant, Hemoglobin A1c increased with EO exposure, whereas fasting insulin and HOMA-IR decreased with EO exposure, and these were most pronounced in the Q4 group. Further to this, there was also a higher prevalence of prediabetes and diabetes in the high-exposure group (Q4), which was accompanied by higher values of white blood cells, neutrophils, monocytes, and GGT. Hypertension prevalence, hemoglobin, triglycerides, and TC/HDL-C were not statistically significant in the log2-HbEO quartile subgroups. It was noted that those diagnosed with prediabetes and diabetes had a higher prevalence of hypertension, higher levels of triglycerides, TC/HDL-C, and HbEO, as well as increased markers of inflammation and oxidative stress (e.g., neutrophil counts, ALP, and GGT), which were more prevalent in males, older ones, and obese individuals.

**Table 1:** Baseline characteristics of the study population based on quartiles of log2-HbEO

**Characteristics**	**Q1 (3.19–4.10)**	**Q2 (4.10–4.49)**	**Q3 (4.49–5.26)**	**Q4 (5.27–10.51)**	***P-value***
Number of participants	799	798	802	801	
**Demographics**					
Age, year	45.43 ± 21.77	45.12 ± 22.66	45.06 ± 21.04	45.83 ± 17.43	*0.877*
Gender, male	361 (45.2%)	394 (49.4%)	345 (43.0%)	485 (60.6%)	*<0.001*
BMI, kg/cm^2^	29.72 ± 7.72	28.87 ± 7.35	28.83 ± 7.32	28.15 ± 7.18	*<0.001*
Poverty income ratio	2.78 ± 1.66	2.69 ± 1.65	2.67 ± 1.61	2.02 ± 1.48	*<0.001*
**Race and ethnicity**					*<0.001*
Mexican American	111 (13.9%)	134 (16.8%)	117 (14.6%)	57 (7.1%)	
Non-Hispanic White	345 (43.2%)	276 (34.6%)	211 (26.3%)	240 (30.0%)	
Non-Hispanic Black	158 (19.8%)	152 (19.0%)	208 (25.9%)	304 (38.0%)	
Other	185 (23.2%)	236 (29.6%)	266 (33.2%)	200 (25.0%)	
**Education**					*0.019*
Less than high school	99 (12.4%)	121 (15.2%)	114 (14.2%)	177 (22.1%)	
High school grad or equivalent	141 (17.6%)	134 (16.8%)	150 (18.7%)	252 (31.5%)	
Some college or above	401 (50.2%)	363 (45.5%)	380 (47.4%)	316 (39.5%)	
**Smoking status**					*<0.001*
Never	487 (61.0%)	456 (57.1%)	500 (62.3%)	179 (22.3%)	
Former	193 (24.2%)	194 (24.3%)	156 (19.5%)	110 (13.7%)	
Current	1 (0.1%)	7 (0.9%)	24 (3.0%)	475 (59.3%)	
**Alcohol consumption**					*0.073*
Nondrinkers	179 (22.4%)	204 (25.6%)	208 (26.0%)	156 (19.5%)	
Moderate	391 (49.0%)	379 (47.5%)	392 (48.9%)	444 (55.4%)	
Hazardous	75 (9.4%)	31 (3.9%)	29 (3.6%)	113 (14.1%)	
**Complete blood count**					
White blood cells, 10^3^ cells/µL	6.99 ± 3.09	7.02 ± 2.12	6.95 ± 2.15	7.61 ± 2.47	*<0.001*
Neutrophils, 10^3^ cells/µL	4.03 ± 1.52	4.06 ± 1.64	3.98 ± 1.56	4.45 ± 1.98	*<0.001*
Lymphocyte, 10^3^ cells/µL	2.16 ± 2.36	2.16 ± 0.87	2.18 ± 0.98	2.30 ± 0.86	*0.151*
Monocyte, 10^3^ cells/µL	0.55 ± 0.18	0.57 ± 0.19	0.56 ± 0.19	0.60 ± 0.22	*<0.001*
**Biochemistry**					
Fasting glucose, mmol/L	6.15 ± 1.98	6.20 ± 1.72	6.20 ± 1.91	6.23 ± 2.50	*0.960*
Fasting insulin, µU/mL	10.38 (6.50–17.64)	10.68 (6.38–16.52)	10.75 (6.30–17.43)	8.17 (5.13–13.80)	*<0.001*
Hemoglobin A1c, %	5.54 ± 0.83	5.75 ± 0.94	5.82 ± 1.09	5.87 ± 1.09	*<0.001*
HOMA-IR	2.71 (1.62–4.85)	2.82 (1.59–4.67)	2.87 (1.62–5.11)	2.10 (1.28–3.77)	*<0.001*
Alkaline phosphatase, IU/L	88.26 ± 51.70	93.94 ± 60.34	91.28 ± 54.02	80.21 ± 31.42	*<0.001*
Gamma glutamyl transferase, IU/L	18.00 (12.00–28.00)	18.00 (13.00–28.00)	19.00 (13.00–28.00)	22.00 (15.00–33.00)	*<0.001*
Hemoglobin, g/dL	13.87 ± 1.62	13.90 ± 1.49	13.78 ± 1.56	14.25 ± 1.48	*0.238*
Triglycerides, mg/dL	102.75 ± 65.82	108.02 ± 73.39	109.89 ± 78.25	104.99 ± 86.72	*0.324*
TC/HDL-C ratio	3.51 ± 1.17	3.54 ± 1.16	3.58 ± 1.22	3.61 ± 1.35	*0.153*
Hypertension	337 (42.2%)	319 (40.0%)	335 (41.8%)	331 (41.3%)	*0.662*
Prediabetes mellitus	257 (32.2%)	296 (37.0%)	288 (35.9%)	318 (39.7%)	*0.017*
Diabetes mellitus	103 (12.9%)	123 (15.4%)	128 (16.0%)	132 (16.5%)	*0.023*

Data are presented as n (%), mean ± standard deviation or median (IQR). **Abbreviations:** BMI, body mass index; HOMA-IR, homeostatic model assessment of insulin resistance; TC/HDL-C, total cholesterol/high-density lipoprotein cholesterol.

### 2. Logistic regression modeling of EO exposure with prediabetes and diabetes

To assess the potential links between EO exposure and the prevalence of prediabetes and diabetes, we constructed univariate and multivariate regression models by logistic regression to analyze the independent effects of log2-HbEO on prediabetes and diabetes. The odds ratio (OR) and 95% confidence intervals are displayed in Table 2. In the full model (Model II) after adjusting for all covariates, log2-HbEO showed an association with an increased prevalence of prediabetes and diabetes, which would be more evident with greater EO exposure. For each one pmol/g Hb, one SD, or two-fold SD increase in log2-HbEO, the risk of prediabetes increased by 12% (1.12, 95% CI 1.04, 1.21), 16% (1.16, 95% CI 1.04, 1.30), and 33% (1.33, 95% CI 1.02, 1.69), with an increased risk of diabetes by 18% (1.18, 95% CI 1.05, 1.37), 26% (1.26, 95% CI 1.07, 1.52), and 61% (1.61, 95% CI 1.14, 1.93), respectively. After converting log2-HbEO from a continuous variable to a categorical variable (quartiles) into the adjusted model, we found that the trend in effect sizes was non-isotropic across the different groups, especially for diabetes. Based on these changes, there may be a nonlinear relationship between log2-HbEO with prediabetes and diabetes.

**Table 2:** The effect of log2-HbEO on prediabetes and diabetes in different models

**Exposure**	**Crude mode (OR, 95%CI, *P*)**	**Mode I (OR, 95%CI, *P*)**	**Mode II (OR, 95%CI, *P*)**
**Prediabetes (n = 1159)**			
Log2-HbEO, pmol/g Hb	1.06 (1.01, 1.12) *0.017*	1.08 (1.02, 1.14) *0.033*	1.12 (1.04, 1.21) *0.036*
Log2-HbEO, pmol/g Hb (per **SD** increment)	1.09 (1.01, 1.17) *0.017*	1.10 (1.02, 1.17) *0.033*	1.16 (1.04, 1.30) *0.036*
Log2-HbEO, pmol/g Hb (per **two-fold SD** increment)	1.17 (1.01, 1.36) *0.017*	1.19 (1.02, 1.37) *0.033*	1.33 (1.02, 1.69) *0.036*
Q1 (3.19–4.10)	Ref	Ref	Ref
Q2 (4.10–4.49)	1.24 (1.01, 1.53) *0.039*	1.26 (1.04, 1.56) *0.035*	1.32 (1.02, 1.66) *0.032*
Q3 (4.49–5.26)	1.18 (1.03, 1.45) *0.034*	1.24 (1.02, 1.54) *0.031*	1.18 (1.09, 1.49) *0.028*
Q4 (5.27–10.51)	1.39 (1.13, 1.70) *0.002*	1.41 (1.17, 1.81) *0.003*	1.53 (1.11, 2.02) *0.008*
*P* for trend	*0.008*	*0.015*	*0.027*
**Diabetes (n = 486)**			
Log2-HbEO, pmol/g Hb	1.08 (1.01, 1.16) *0.025*	1.11 (1.03, 1.19) *0.004*	1.18 (1.05, 1.37) *0.009*
Log2-HbEO, pmol/g Hb (per **SD** increment)	1.11 (1.01, 1.22) *0.025*	1.15 (1.05, 1.27) *0.004*	1.26 (1.07, 1.52) *0.009*
Log2-HbEO, pmol/g Hb (per **two-fold SD** increment)	1.24 (1.03, 1.49) *0.025*	1.33 (1.10, 1.62) *0.004*	1.61 (1.14, 1.93) *0.009*
Q1 (3.19–4.10)	Ref	Ref	Ref
Q2 (4.10–4.49)	1.26 (0.95, 1.67) *0.111*	1.27 (0.96, 1.79) *0.114*	1.31 (0.95, 1.87) *0.133*
Q3 (4.49–5.26)	1.13 (1.05, 1.51) *0.014*	1.19 (1.04, 1.57) *0.018*	1.24 (1.03, 1.80) *0.025*
Q4 (5.27–10.51)	1.36 (1.03, 1.80) *0.030*	1.50 (1.12, 2.01) *0.007*	1.60 (1.17, 2.32) *0.009*
*P* for trend	*0.068*	*0.015*	*0.191*

The risks of prediabetes and diabetes are both relative to the euglycemia population (n = 1555). **Crude Model** adjusted for none. **Model I** adjusted for age and gender. **Model II** adjusted for age, gender, BMI, poverty income ratio, race, education, smoking status, alcohol consumption, complete blood count, alkaline phosphatase, gamma glutamyl transferase, hemoglobin, triglycerides, TC/HDL-C ratio, and hypertension. **Abbreviations:** SD, standard deviation, equal to 1.38 in this study.

### 3. Dose-response relationships, threshold effects, and changes in indicators of impaired glucose metabolism

**Fig. 1:**
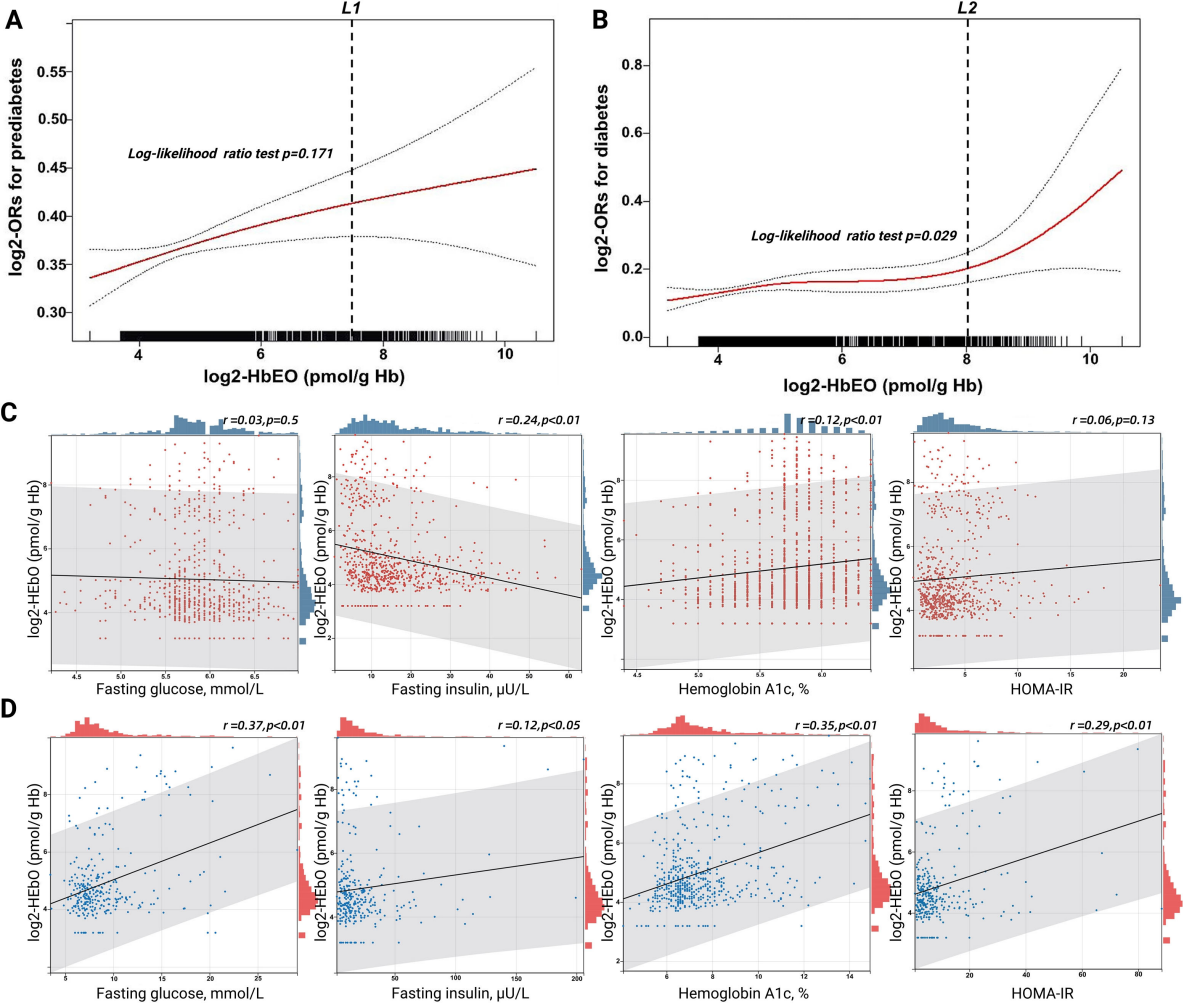
Dose-response relationship and correlation analysis between EO exposure and impaired glucose metabolism. **A.** Linear relation between log2-HbEO and prediabetes in a generalized additive model (GAM). The solid red line represents the smooth curve fit between variables. Blue bands represent the 95% confidence interval from the fit. The dashed line L1 represents the threshold inflection point (log2-HbEO = 7.52), and log-likelihood ratio test p > 0.05 indicates that constructing a two-piecewise linear regression on either side of the inflection point is inferior to the standard linear regression fitted model. Model adjusted for age, gender, BMI, poverty income ratio, race, education, smoking status, alcohol consumption, complete blood count, alkaline phosphatase, gamma-glutamyl transferase, hemoglobin, triglycerides, TC/HDL-C ratio, and hypertension. **B.** J-shaped relation between log2-HbEO and diabetes in GAM. L2 represents the inflection point (log2-HbEO = 8.03), and log-likelihood ratio test p < 0.05 indicates that the two-piecewise linear regression model is more appropriate. Model was adjusted same as Fig. 1A. **C.** Pearson correlation of log2-HbEO with indicators of impaired glucose metabolism in prediabetic individuals. Red dots represent scatter distributions, blue bars represent data distributions, and black lines represent correlation trends. The gray area represents r’s 95% CI, r > 0 indicates positive correlation, r < 0 indicates negative correlation, and p < 0.05 is considered statistically significant. **D.** Pearson correlation of log2-HbEO with indicators of impaired glucose metabolism in diabetic individuals. Blue dots represent scatter distributions, red bars represent data distributions, and others same as Fig. 1C. **Abbreviations:** OR, odds ratio. HOMA-IR, homeostatic model assessment of insulin resistance.

We assessed the dose-response relationships between log2-HbEO with the prevalence of prediabetes and diabetes via fitting an additive generalized model and penalized spline approach (Fig. 1 and Table 3). Smoothed curve results showed that log2-HbEO was correlated with prediabetes in an approximately linear manner. Although the threshold inflection point for prediabetes was drawn as log2-HbEO = 7.52 (pmol/g Hb), the P > 0.05 for the log-likelihood ratio test indicated that the fitting model by standard linear regression was more representative. In contrast, log2-HbEO showed a “J”-shaped trend with diabetes risk, and the fitting model by two-piecewise linear regression was more appropriate due to the P = 0.029 for the likelihood ratio test. The recursive algorithm further premeditated the diabetes threshold inflection point to be log2-HbEO = 8.03 (pmol/g Hb), with an OR and 95% CI of 1.11 (1.01–1.19) on the left side of the point as well as 2.10 (1.08–4.06) on the right side, which were both statistically significant (p < 0.05). The model showed a positive correlation between the prevalence of prediabetes and diabetes along with increased log2-HbEO levels after adjusting for all covariates.

**Table 3:** The results of the two-piecewise linear model

**Exposure**	**Prediabetes (n = 1159)**		**Diabetes (n = 486)**	

	**OR (95%CI)**	***P-value***	**OR (95%CI)**	***P-value***
**Fitting model by standard linear regression**				
Log2-HbEO, pmol/g Hb	1.12 (1.04, 1.21)	*0.036*	1.18 (1.05, 1.37)	0.009
Log2-HbEO, pmol/g Hb (per SD increment)	1.16 (1.04, 1.30)	0.036	1.26 (1.07, 1.52)	0.009
Log2-HbEO, pmol/g Hb (per two-fold SD increment)	1.33 (1.02, 1.69)	0.036	1.61 (1.14, 2.30)	0.009
**Fitting model by two-piecewise linear regression**				
Inflection point of log2-HbEO, pmol/g Hb	7.52		8.03	
Left of the point	1.12 (1.01, 1.24)	*0.030*	1.11 (1.01, 1.19)	*0.025*
Right of the point	1.30 (0.95, 1.23)	*0.083*	2.10 (1.08, 4.06)	*0.033*
Log-likelihood ratio test		*0.171*		*0.029*

The risks of prediabetes and diabetes are both relative to the euglycemia population (n = 1555). Model was adjusted for age, gender, BMI, poverty income ratio, race, education, smoking status, alcohol consumption, complete blood count, alkaline phosphatase, gamma glutamyl transferase, hemoglobin, triglycerides, TC/HDL-C ratio, and hypertension. **Abbreviations:** SD, standard deviation, equal to 1.38 in this study.

In addition, we analyzed trends in indicators of impaired glucose metabolism accompanying EO exposure using Pearson’s correlation during the same period (Fig. 1C and 1D). The results revealed that EO exposure was associated with reduced fasting insulin and elevated HbA1c in the prediabetic stage. While in the diabetes stage, EO exposure was correlated with elevated fasting glucose, HbA1c, and HOMA-IR, suggesting an exacerbation of diabetes progression by EO exposure. Due to the exogenous injection of long-acting insulin, the association of EO exposure with fasting insulin in diabetic individuals was perturbed.

### 4. Subgroup analysis and interactions

**Fig. 2:**
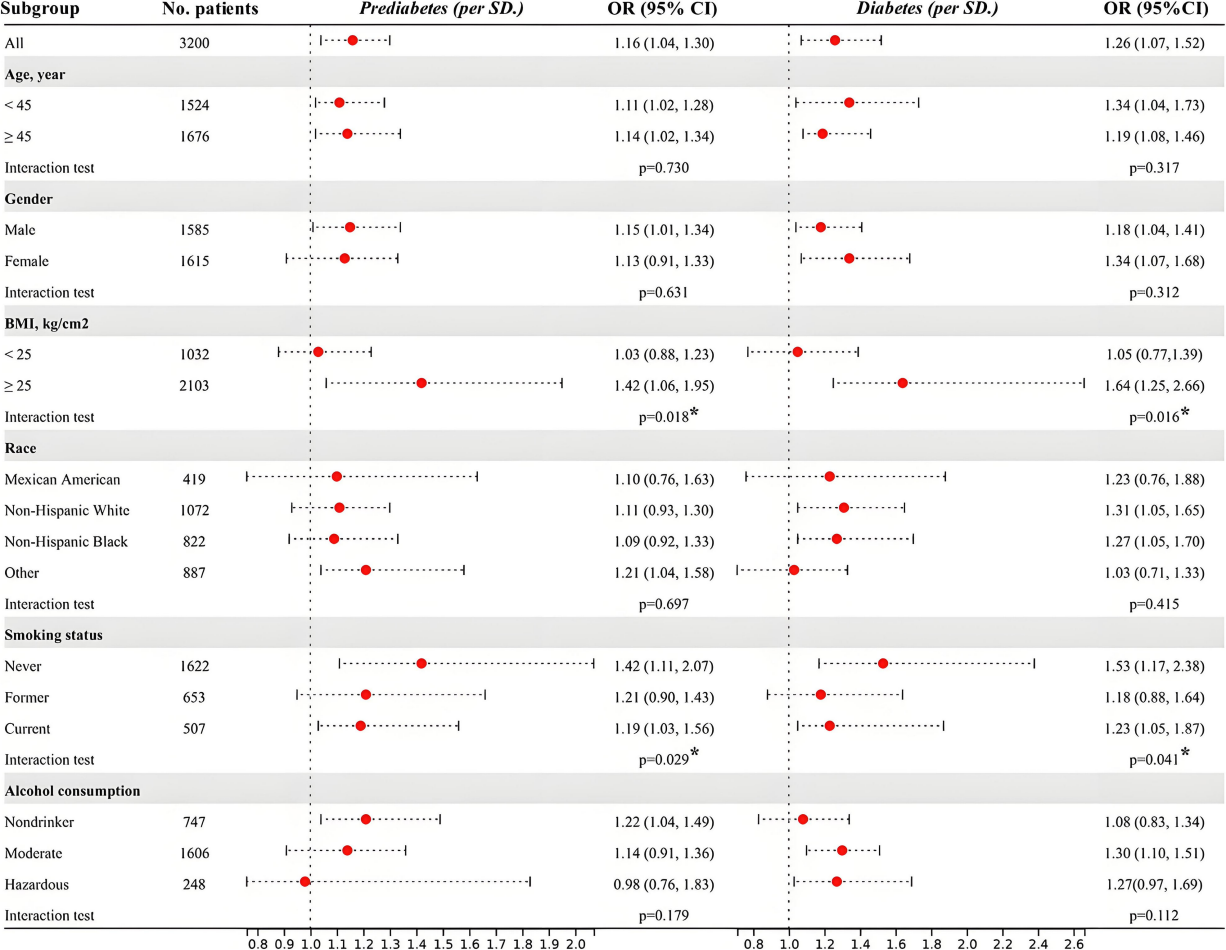
Subgroup analysis and interactions of per SD increment on the risk of prediabetes and diabetes. Model was adjusted for all covariates. *P values indicate an interaction with EO exposure. **Abbreviations:** SD, standard deviation, equal to 1.38 in this study.

As shown in Fig. 2, the positive association of ethylene oxide exposure in the prevalence of prediabetes and diabetes was consistent within age subgroups. No significant interactions were observed in subgroups stratified by age, gender, race, education, and drinking status (P value for interaction > 0.05). Obese people (BMI > 25) may be more susceptible to EO exposure-related prediabetes and diabetes compared to non-obese ones (P-value for interaction < 0.05). In addition, elevated HbEO levels in never-smokers predicted a higher risk of disease associated with EO exposure (p-value for interaction < 0.05), which suggested a potential difference between EOs from tobacco smoke and those from other routes of exposure.

### 5. Mediating effects of inflammation and oxidative stress

**Fig. 3:**
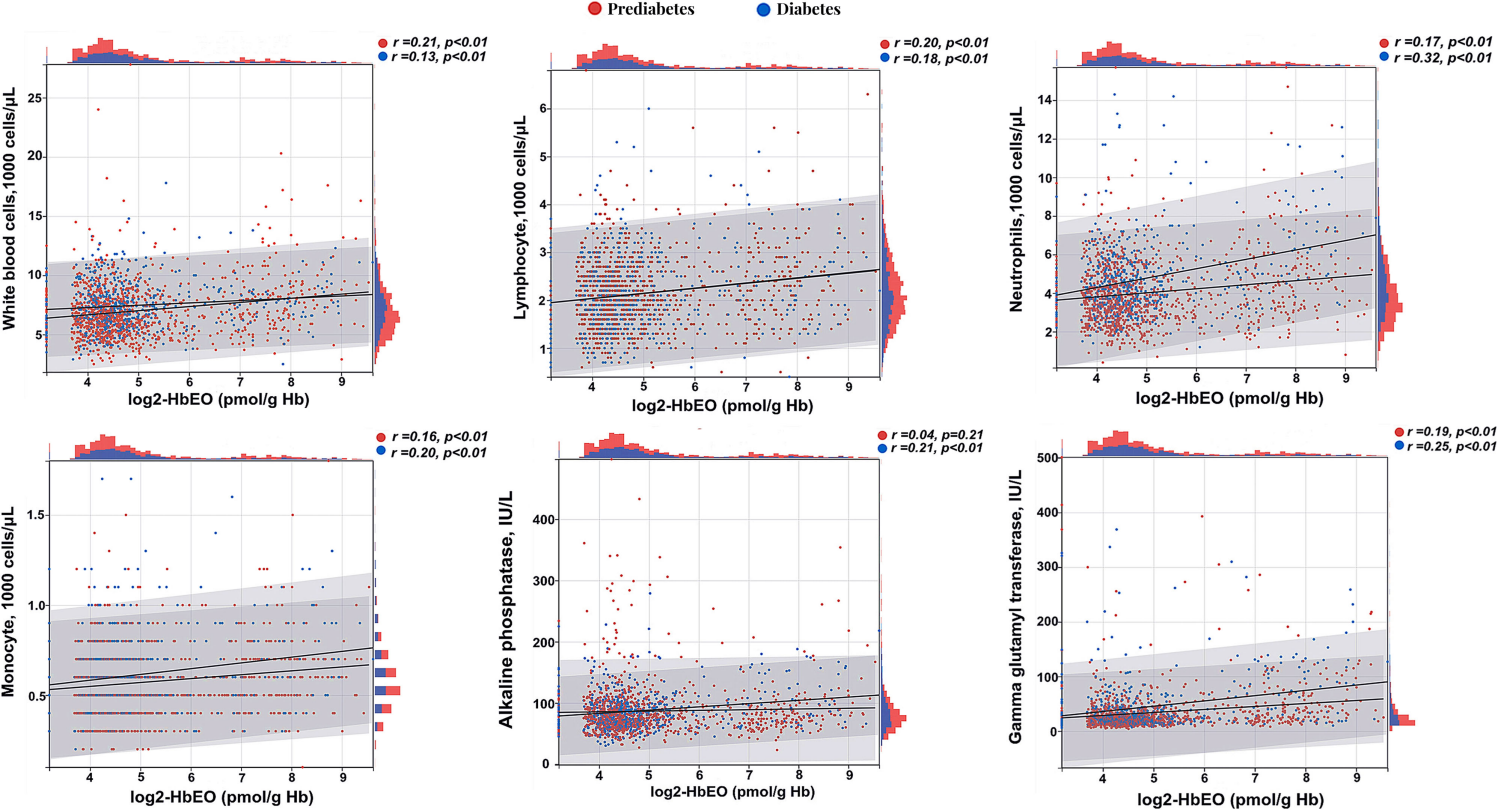
Pearson correlation of log2-HbEO with mediators in different states of impaired glucose metabolism. Red and blue dots represent scatter distributions for prediabetic and diabetic individuals, respectively, bands represent the corresponding data distributions, and black lines represent correlation trends. The gray area represents r’s 95% CI, r > 0 indicates positive correlation, r < 0 indicates negative correlation, and p < 0.05 is considered statistically significant.

Our results showed that there was an association between EO exposure and changes in the levels of inflammatory and oxidative stress biomarkers in vivo, including WBC, lymphocytes, monocytes, neutrophils, and GGT (absolute value of β > 0, P < 0.05) (Supplementary Table S2). These biomarkers were also related to the prevalence of prediabetes and diabetes (Supplementary Table S3). Based on these findings, we introduced mediating variables (inflammation and oxidative stressors) with EO exposure as the independent variable, and when prediabetes and diabetes were the dependent variables, we found that these moderators mediated a portion of the effect between EO exposure and impaired glucose metabolism (Table 4). The results revealed that inflammatory and oxidative stress markers (monocytes, neutrophils, and GGT) mediated the links between EO exposure and prediabetes. Among them, monocytes had the most significant mediating effect, with a mediated proportion of 8.21%. Meanwhile, monocytes, neutrophils, ALP, and GGT mediated the relationship between EO and diabetes. Neutrophils had the most significant mediating effect, with a mediated proportion of 8.72%. Mediation analysis performed with (EO → prediabetes/diabetes → inflammation and oxidative stress) as an alternative pathway was not statistically significant (shown in Table S4). Pearson’s correlation analysis was applied to examine the relations between log2-HbEO with these biomarkers at different stages of glucose metabolic dysfunction (Fig. 3). The results showed a corresponding increment of inflammatory and oxidative stress biomarkers with increasing EO exposure in individuals with impaired glucose metabolism, which showed consistent findings with mediation analysis.

**Table 4:** Mediating effect and proportions of biomarkers between EO exposure with prediabetes and diabetes

**Outcomes**	**Mediators**	**Indirect effect, e-04**	**95% CI, e-04**	**Proportion, %**	***P-value***
Prediabetes	White blood cell	15.13	−9.40, 57.30	3.55	*0.120*
	Lymphocyte	1.15	−7.33, 3.26	0.27	*0.721*
	Monocyte	35.00	23.76, 99.73	8.21	*0.000*
	Neutrophils	17.99	1.88, 34.73	4.22	*0.011*
	Alkaline phosphatase	4.05	−1.91, 12.77	0.95	*0.369*
	Gamma glutamyl transferase	13.30	2.21, 34.52	3.12	*0.000*
Diabetes	White blood cell	25.03	−7.12, 38.26	4.00	*0.396*
	Lymphocyte	10.57	−3.93, 20.37	1.69	*0.265*
	Monocyte	13.77	2.02, 27.49	2.20	*0.004*
	Neutrophils	54.57	6.32, 87.33	8.72	*0.001*
	Alkaline phosphatase	12.27	3.83, 24.71	1.96	*0.002*
	Gamma glutamyl transferase	18.59	1.99, 28.76	2.97	*0.008*

Model was adjusted for age, gender, BMI, poverty income ratio, race, education, smoking status, alcohol consumption, complete blood count, alkaline phosphatase, gamma glutamyl transferase, hemoglobin, triglycerides, TC/HDL-C ratio, and hypertension.

## Discussion

People are commonly exposed to compounds such as EOs daily with a cumulative health-damaging effect, which would be magnified indefinitely with the worsening of global environmental pollution [25]. So, there has been a continuing public concern about how to reveal dose-effect relations between pollutant exposures and human diseases. In this survey, we observed that EO exposure was associated with impaired glucose metabolism, as evidenced by a link with changes in glucose metabolic indicators and an increase in the prevalence of prediabetes and diabetes, and this positive trend was consistent across age subgroups. Subsequent dose-response curves revealed that this positive correlation was approximately linear with prediabetes and “J” shaped with diabetes. When log2-HbEO > 8.03 (HbEO > 261.38 pmol/g Hb), the risk of diabetes would be further increased, which provides a reference value for the exposed and diabetes risk groups for health and occupational management. In addition, a potential mechanism is that the early stages of impaired glucose metabolism may be initiated by EO-related inflammation and oxidative stress damaging pancreatic β-cells, resulting in decreased insulin secretion. These speculations were partially supported by mediation analysis and Pearson analysis. The robustness of these findings was also demonstrated in various sensitivity and stratified analyses.

In recent decades, increasing evidence has revealed the tendency of EO exposure to drive multiple diseases such as cancer [5], adverse reproductive outcome [30], neurotoxicity [7], hypertension [20], cardiovascular disease [18], and metabolic syndrome [19]. Hogstedt et al. obtained the earliest epidemiological evidence linking EO chronic exposure to malignancy via a long-term follow-up of occupationally exposed workers [31]. A series of subsequent experiments in vivo and in vitro also confirmed this high carcinogenic risk, improving EO regulation during production or usage [1]. Godderis et al. reported dose-dependent DNA damage induced by EOs, revealing the mechanism of exposure-related genotoxicity [32]. Two years earlier, Guo et al. presented a report on the increased prevalence of diabetes associated with EO exposure during NHANES 2013–2016, further corroborating the positive correlation of EO exposure with glucose metabolism dysfunction [21]. Consistent with previous results, HbA1c levels in our present study increased following the EO exposure values. Differently, fasting insulin levels and HOMA-IR showed a negative trend with EO exposure, which may suggest a potential mechanism whereby EO exposure impacts insulin secretion by pancreatic β-cells rather than promoting insulin resistance. Combining the results of mediation and Pearson correlation analyses, we propose the hypothesis of “EO → inflammation and oxidative stress → disordered glucose metabolism”. This alteration of β-cell function may occur in the early stages of diabetes development (prediabetes) and progressively worsen with the disease. This health-damaging effect may be caused on the one hand by an increase in reactive oxygen species (ROS) and chronic inflammation induced by EO itself [9, 33]. On the other hand, it may also occur via the toxic effects of EO metabolites such as ethylene chlorohydrin and ethylene glycol [34, 35]. Although this hypothesis may be unilateral and uncertain, it provides a possible explanation for the pathogenic mechanism of EO exposure and impaired glucose metabolism. Additionally, EO can also cause epigenetic modifications such as DNA methylation and histone modification, which in conjunction with overnutrition, inflammation, and other types of stress collaboratively disrupt the homeostatic epigenetic signatures in β-cells. Dysregulated epigenetic signatures, and the associating transcriptional outputs, lead to the dysfunction and eventual loss of β-cells [6, 36].

The National Diabetes Statistics Report provides a prediabetes prevalence of 38% and a diabetes prevalence of 11.6% in the overall U.S. population between 2017 and 2020, including higher rates in obese individuals [37]. This report is roughly consistent with our findings, suggesting favorable reliability and representativity of our samples. Diabetes is a disorder of glucose regulation due to insufficient insulin secretion and insulin resistance, in which inflammation and oxidative stress also have long been considered the main components of pancreatic β-cell failure. Relevant pathophysiology is well described in previous reviews, such as chronic inflammation, antioxidant imbalance, immune dysregulation, and DNA damage [36, 38]. The dose-response relationship indicated that the risk of impaired glucose metabolism was further raised after exposure to a certain dose of EOs. Based on our hypothesis, effectively suppressing inflammatory and oxidative stress imbalance may be a potential way to reduce glucose metabolism abnormalities caused by EO exposure [27]. When combined with genetic susceptibility, such a dose response facilitates identifying individuals at increased risk for diabetes as well as early intervention [13].

Interestingly, subgroup analyses found that the risk of abnormal glucose metabolism from EO exposure was more significant in the never-smoking individuals, whereas no positive trend was observed in the smoking population. This similar interaction is also found in other EO exposure-related disease studies, but no rational explanation has been given. Based on the Center for Disease Control 2019 NHANES (Combined 2013–2016), smoking significantly increases HbEO levels compared to nonsmoking populations (mean HEV levels, 236 vs. 31.4 pmol/g Hb) [3, 39]. On the one hand, we speculate that this increasing effect may interfere with the assessment of disease risk from other pathways of EO exposure in the environmental context, such as occupational exposure, air exposure, and endogenous production [3, 40–42]. On the other hand, nonsmokers would need more environmental EO exposures, such as occupational exposures, at the same pathogenic level of HbEO (e.g., 300 pmol/g Hb) compared to smokers. Such higher exposures result in more EO-related inflammation, reactive oxygen species, lipid peroxidation, DNA damage, and epigenetic modifications in vivo, which may disrupt pancreatic β-cell function, leading to a progressive decline in insulin secretion and glucose tolerance. However, current evidence generally agrees that smoking is a risk factor for diabetes, and in particular that nicotine in tobacco has been found to decrease glucose tolerance, lower insulin secretion, and increase insulin resistance and glucagon secretion [43, 44]. We have to be honest and admit that these effects seem to run somewhat counter to our subgroup findings, and we are not sure whether the increased risk of diabetes from smoking is related to EO exposure in tobacco. These doubts were difficult to confirm in our study. Although the biological mechanisms underlying the smoking-diabetes relation could be similar among different populations, differences still exist in smoking patterns, its correlations with genetic susceptibility to diabetes, population characteristics, and other lifestyle behaviors [45]. We hope to obtain more evidence in future studies for the different impacts of EO exposure on glucose metabolism in nonsmokers and smokers.

Compared with previous reports, our study supplemented more analyses about the risk of prediabetes and diabetes, such as dose-response relations, subgroup analyses, and mediation analyses. These findings increased the possibility of explaining the association between EO exposure and impaired glucose metabolism in the general population. Based on a meta-analysis of available evidence, Kirman and Hays in 2017 proposed an endogenous equivalent air exposure equation for EO [46]. We calculated the corresponding air EO exposure levels based on the equation, i.e., the overall population mean HbEO was 31.56 pmol/g Hb, equivalent to 2.90 ppb air exposure; the log2-HbEO inflection point for prediabetes was 7.52 pmol/g Hb, equivalent to 16.84 ppb; and for diabetes was 8.03 pmol/g Hb, equivalent to 23.98 ppb. According to the U.S. Environmental Protection Agency’s (USEPA) 2019 published monitoring report for EO in ambient air, the mean range of air EO concentrations in rural and urban areas of the U.S. is 0.16 to 0.17 ± 0.04 ppb [3]. Even near plant emission sources, the weighted mean air EO concentrations are only 0.33 ± 0.76 ppb and 0.20 ± 0.55 ppb. These monitoring results suggest that routine ambient air EO exposure is not sufficient to induce EO-related impairment of glucose metabolism due to the difference in at least two orders of magnitude. However, according to Filser and Klein (2018) regarding occupational EO exposure, mean HbEO levels were estimated between 720 and 5000 pmol/g Hb, corresponding to daily (8 h/day, 5 days/week) exposures to 1 ppm (= 1000 ppb) at steady state [47]. Such mean HbEO levels are above the inflection point for impaired glucose metabolism in our study. Therefore, we propose that continued occupationally EO high exposure may increase the risk of impaired glucose metabolism.

The strength of this study is the use of a population-based and quality-controlled large database, and the prevalence associated with EO exposure presented in a stepwise manner. The robustness and reliability of the results were also enhanced by using multiple statistical methods and adjusting for potential confounding variables. However, there are still some inherent limitations that need to be addressed. First, due to the cross-sectional design, we could not establish a causal link between EO exposure and individuals with impaired glucose metabolism. Second, owing to the absence of diabetes type enrollment in the NHANES database, we could not identify whether members had type 1 or type 2 diabetes, which needs more evidence to explore the effect of EO exposure on diabetes types. Finally, due to the lack of occupational classification information and genetic history of diabetes for members in the NHANES (2017–2020) data, we were unable to consider the impact of whether members were occupationally exposed and their potential genetic susceptibility. Due to limited data, the effects of temperature and pH during the testing period on HbEO were also not considered for the time being [48], as well as the difference between the hazards of short- or long-term exposure. It is expected that future studies will provide relevant experimental evidence.

Our findings may provide some insights for susceptible populations who may need to focus more on occupational protection, healthy diet, lifestyle management, and diabetes screening. These correlated risks also provide a potential measure for disease decision-making that could contribute to global diabetes governance. With improved regulations during production and usage, as well as reduced environmental problems, we believe that diseases associated with EO exposure will be effectively managed.

## Conclusion

Elevated ethylene oxide exposure increases the incidence of impaired glucose metabolism in the general U.S. population, and the potential mechanisms may be mediated by inflammation and oxidative stress. More prospective evidence should be needed in the future to confirm these findings.

## Supplementary information

**Additional file 1:** **Table S1.** Baseline characteristics of population based on impaired glucose metabolism. **Table S2.** Association of ethylene oxide exposure with inflammation and oxidative stress. **Table S3.** The effects of inflammation and oxidative stress biomarkers on the prevalence of prediabetes and diabetes. **Table S4.** Mediation analysis performed with (EO → prediabetes/diabetes → inflammation and oxidative stress) as an alternative pathway. **Figure S1.** Flowchart of participants included in this study. NHANES, National Health and Nutrition Examination Survey; HbA1C, hemoglobin A1c.

## Declarations

### Availability of data and material

The datasets used for these analyses are publicly available (https://www.cdc.gov/nchs/nhanes/index.htm), which could be provided as required.
