# Supplementary material for: Ethylene oxide exposure increases impaired glucose metabolism in the US general population: a national cross-sectional study
Source: Environ Health Prev Med. 2024 Dec 7;29:68. doi: 10.1265/ehpm.24-00199 (PMC11631556; doi:10.1265/ehpm.24-00199)
Supplement: Supplementary file 1 — Additional file 1: Table S1. Baseline characteristics of population based on impaired glucose metabolism. Table S2. Association of ethylene oxide exposure with inflammation and oxidative stress. Table S3. The effects of inflammation and oxidative stress biomarkers on the prevalence of prediabetes and diabetes. Table S4. Mediation analysis performed with (EO → prediabetes/diabetes → inflammation and oxidative stress) as an alternative pathway. Figure S1. Flowchart of participants included in this study. NHANES, National Health and Nutrition Examination Survey; HbA1C, hemoglobin A1c. [file ehpm-29-068-s001.docx]

**Supplementary materials**

**Table S1. Baseline characteristics of population based on impaired glucose metabolism**

| **Characteristics** | **Total** | **Euglycemia** | **Prediabetes** | **Diabetes** | ***P- value*** |
| --- | --- | --- | --- | --- | --- |
| Number of participants | 3200 | 1555 | 1159 | 486 |  |
| **Demographics** |  |  |  |  |  |
| Age, year | 45.36 ± 20.81 | 37.27±19.87 | 52.36 ± 19.05 | 59.01 ± 14.48 | <0.001 |
| Gender, male | 1585 (49.53%) | 717 (46.11%) | 619 (53.41%) | 249 (51.23%) | <0.001 |
| BMI, kg/cm^2^ | 28.89 ± 7.41 | 27.16 ± 6.85 | 30.11 ± 7.25 | 32.62 ± 7.98 | <0.001 |
| Poverty income ratio | 2.54 ± 1.63 | 2.57 ± 1.64 | 2.56 ± 1.63 | 2.38 ± 1.56 | 0.611 |
| Hypertension, % | 1322 (41.31%) | 615 (39.55%) | 487 (42.02%) | 223(45.27%) | 0.028 |
| **Race** |  |  |  |  | 0.029 |
| Mexican American | 511 (19.30%) | 303 (19.49%) | 138 (11.91%) | 70 (14.40%) |  |
| Non-Hispanic White | 1072 (33.50%) | 561 (36.08%) | 372 (32.10%) | 139 (28.60%) |  |
| Non-Hispanic Black | 822 (25.69%) | 351 (22.57%) | 334 (28.82%) | 137 (28.19%) |  |
| Other | 887 (27.72%) | 449 (28.87%) | 315 (27.18%) | 123 (25.31%) |  |
| **Education** |  |  |  |  | 0.077 |
| Less than high school | 511 (19.30%) | 194 (12.48%) | 203 (19.13%) | 114 (23.47%) |  |
| High school grad or equivalent | 677 (25.57%) | 266 (17.11%) | 289 (27.24%) | 122 (25.10%) |  |
| Some college or above | 1460 (55.14%) | 666 (42.83%) | 569 (53.63%) | 225 (46.30%) |  |
| **Smoking status** |  |  |  |  | 0.038 |
| Never | 1622 (58.30%) | 790 (50.80%) | 598 (55.42%) | 234 (48.15%) |  |
| Former | 653 (23.47%) | 238 (15.31%) | 279 (25.86%) | 136 (27.98%) |  |
| Current | 507 (18.22%) | 213 (16.70%) | 202 (18.72%) | 92 (18.93%) |  |
| **Alcohol consumption** |  |  |  |  | 0.008 |
| Nondrinkers | 747 (28.72%) | 259 (16.66%) | 326 (32.34%) | 162 (33.33%) |  |
| Moderate | 1606 (61.75%) | 772 (49.65%) | 605 (60.02%) | 229 (47.12%) |  |
| Hazardous | 248 (9.53%) | 134 (8.62%) | 77 (7.64%) | 37 (7.61%) |  |
| **Complete blood count** |  |  |  |  |  |
| WBC,10^3^ cells/μL | 7.14 ± 2.50 | 7.10 ± 2.11 | 7.09 ± 3.08 | 7.50 ± 2.13 | 0.446 |
| Neutrophils,10^3^ cells/μL | 4.13 ± 1.69 | 4.12 ± 1.67 | 4.03 ± 1.75 | 4.47 ± 1.66 | 0.021 |
| Lymphocyte,10^3^ cells/μL | 2.20 ± 1.42 | 2.19 ± 0.70 | 2.23 ± 2.16 | 2.16 ± 0.82 | 0.402 |
| Monocyte, 10^3^ cells/μL | 0.57 ± 0.19 | 0.56 ± 0.19 | 0.57 ± 0.20 | 0.61 ± 0.22 | 0.713 |
| **Biochemistry** |  |  |  |  |  |
| Fasting glucose, mmol/L | 6.19 ± 2.04 | 5.50 ± 1.50 | 5.90 ± 0.47 | 8.49 ± 3.41 | <0.001 |
| Fasting insulin (μU/mL) | 9.87 (6.12-16.39) | 8.77 (5.88-11.03) | 11.03 (7.05-16.92) | 14.11(8.59-25.47) | <0.001 |
| Hemoglobin A1c, % | 5.74 ± 1.00 | 5.36 ± 0.63 | 5.74 ± 0.36 | 7.10 ± 1.65 | <0.001 |
| HOMA-IR | 2.62 (1.52-4.61) | 2.07 (1.21-3.73) | 2.88 (1.81-4.68) | 5.33 (2.90-9.39) | <0.001 |
| ALP, IU/L | 88.47 ± 50.86 | 78.94 ± 59.36 | 90.59 ± 41.80 | 89.98 ± 32.42 | 0.013 |
| GGT, IU/L | 19.00 (13.00-30.00) | 13.00 (9.00-27.00) | 21.00 (15.00-31.00) | 25.00 (18.00-43.00) | 0.008 |
| Hemoglobin, g/dL | 13.95 ± 1.55 | 13.95 ± 1.52 | 13.99 ± 1.54 | 13.96 ± 1.66 | 0.877 |
| Triglycerides, mg/dL | 103.85 ± 76.46 | 94.60 ± 56.06 | 107.85 ± 73.52 | 116.43 ± 84.48 | 0.012 |
| TC/HDL-C ratio | 3.48 ± 1.23 | 3.40 ± 1.12 | 3.51 ± 1.21 | 3.73 ± 1.50 | 0.016 |
| HbEO, pmol/g Hb | 22.50 (17.20-38.50) | 17.20 (13.30-33.10) | 23.00 (17.80-43.00) | 23.20(17.90-42.90) | 0.003 |
| Log2-HbEO, pmol/g Hb | 4.98 ± 1.38 | 4.90 ± 1.35 | 5.06 ± 1.42 | 5.11 ± 1.49 | 0.017 |

Data are presented as n (%) , mean ± standard deviation or median (IQR). **Abbreviations:** BMI, body mass index; HOMA-IR, homeostatic model assessment of insulin resistance, WBC, white blood cells; ALP, alkaline phosphatase; GGT, gamma glutamyl transferase; HbEO, hemoglobin adducts of ethylene oxide; TC/HDL-C, total cholesterol/high-density lipoprotein cholesterol.

**Table S2. Association of ethylene oxide exposure with inflammation and oxidative stress.**

|  | **β** | **95% CI** | ***P-value*** |
| --- | --- | --- | --- |
| White blood cells | 0.35 | 0.20, 0.46 | <0.001 |
| Lymphocyte | 0.03 | 0.01, 0.05 | 0.021 |
| Monocyte | 0.09 | 0.02, 0.19 | <0.001 |
| Neutrophils | 0.16 | 0.12, 0.28 | <0.001 |
| Alkaline phosphatase | 0.44 | -0.64, 1.36 | 0.407 |
| Gamma glutamyl transferase | 1.92 | 0.84, 3.08 | 0.011 |

Model adjusted for other covariates such as age, gender, BMI, poverty income ratio, race, education, smoking status, alcohol consumption, hemoglobin, triglycerides, TC/HDL-C ratio, and hypertension.

**Table S3. The effects of inflammation and oxidative stress biomarkers on the prevalence of prediabetes and diabetes.**

|  | **Prediabetes** | | **Diabetes** | |
| --- | --- | --- | --- | --- |
|  | **OR (95% CI)** | ***P-value*** | **OR (95% CI)** | ***P-value*** |
| White blood cells | 1.16 (1.08, 1.28) | 0.001 | 1.23 (0.90, 1.46) | 0.273 |
| Lymphocyte | 1.06 (0.69, 1.33) | 0.382 | 0.93 (0.82, 1.31) | 0.241 |
| Monocyte | 1.20 (1.13, 1.38) | 0.003 | 1.24 (1.04, 1.46) | 0.009 |
| Neutrophils | 1.11 (1.02, 1.29) | 0.015 | 1.36 (1.13, 1.76) | 0.002 |
| Alkaline phosphatase | 0.91 (0.79, 1.16) | 0.355 | 1.07 (1.02, 1.15) | 0.006 |
| Gamma glutamyl transferase | 1.13 (1.05, 1.30) | 0.020 | 1.23 (1.05, 1.48) | 0.003 |

Model adjusted for other covariates such as age, gender, BMI, poverty income ratio, race, education, smoking status, alcohol consumption, hemoglobin, triglycerides, TC/HDL-C ratio, and hypertension.

**Table S4. Mediation analysis performed with (EO → prediabetes/diabetes → inflammation and oxidative stress) as an alternative pathway.**

| Outcomes | Mediators | Indirect effect | 95% CI | Proportion | *P-value* |
| --- | --- | --- | --- | --- | --- |
| White blood cell | Prediabetes | -0.002 | -0.024，0.005 | -0.007 | *0.329* |
|  | Diabetes | 0.003 | 0.000,0.007 | 0.013 | *0.113* |
| Lymphocyte | Prediabetes | 0.001 | -0.001, 0.003 | 0.011 | *0.548* |
|  | Diabetes | -0.001 | -0.002,0.000 | -0.009 | *0.290* |
| Monocyte | Prediabetes | 0.000 | -0.000,0.001 | 0.001 | *0.871* |
|  | Diabetes | 0.000 | 0.000,0.001 | 0.020 | *0.111* |
| Neutrophils | Prediabetes | -0.003 | -0.043,0.002 | -0.017 | *0.074* |
|  | Diabetes | 0.003 | 0.000,0.007 | 0.020 | *0.085* |
| Alkaline phosphatase | Prediabetes | -0.055 | -0.142,0.000 | 0.018 | *0.137* |
|  | Diabetes | -0.026 | -0.079，0.007 | 0.009 | *0.246* |
| Gamma glutamyl transferase | Prediabetes | 0.066 | 0.000，0.189 | 0.077 | *0.186* |
|  | Diabetes | 0.145 | -0.014，0.371 | 0.170 | *0.155* |

Model was adjusted for age, gender, BMI, poverty income ratio, race, education, smoking status, alcohol consumption, complete blood count, alkaline phosphatase, gamma glutamyl transferase, hemoglobin, triglycerides, TC/HDL-C ratio, and hypertension.

**
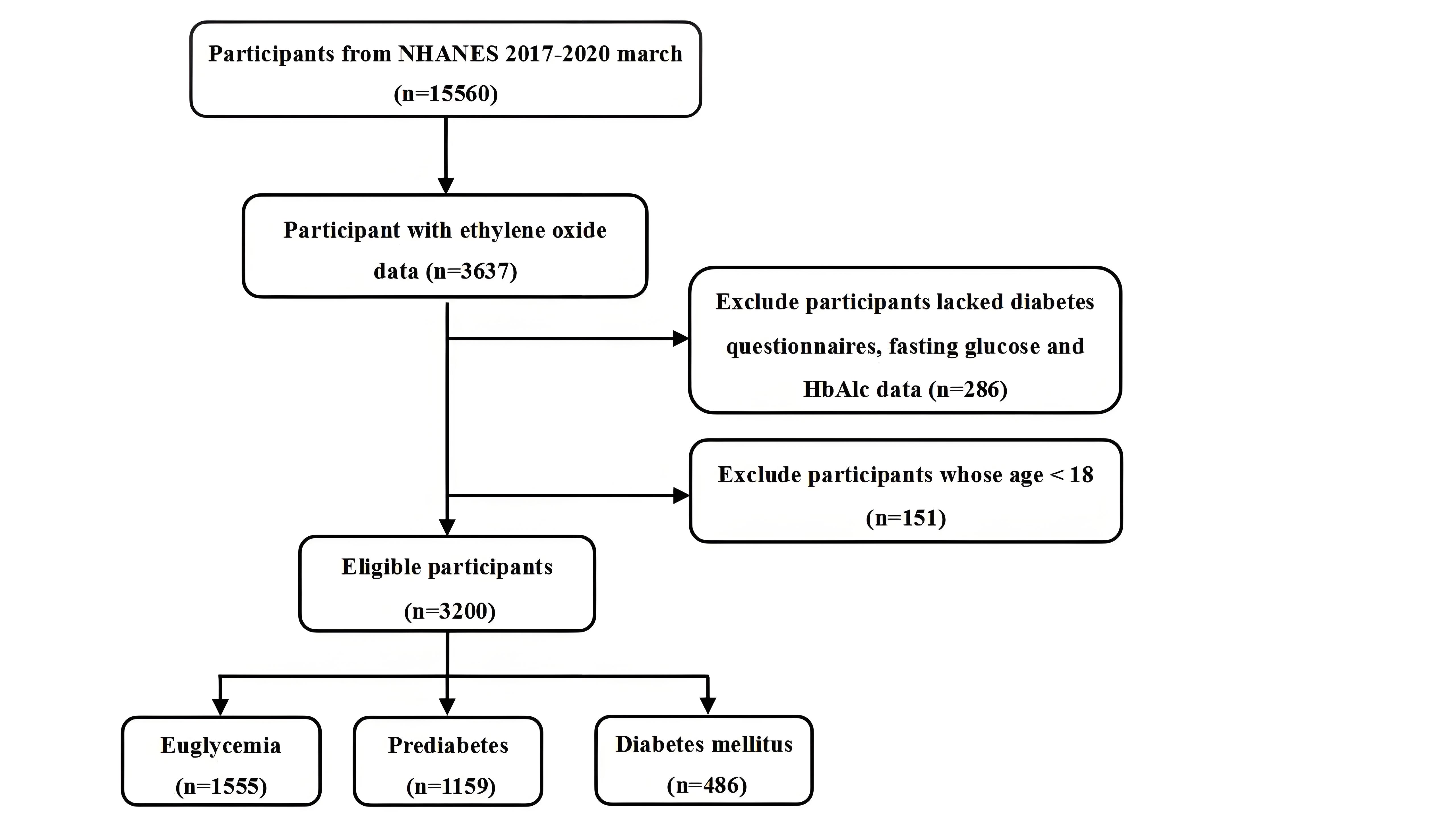
**

**Figure S1**. Flowchart of participants included in this study. NHANES, National Health and Nutrition Examination Survey; HbA1C, hemoglobin A1c.
